# PD17 Next-Generation Sequencing Gene Panel For Gastrointestinal Stromal Tumor Care

**DOI:** 10.1017/S0266462325101529

**Published:** 2025-12-29

**Authors:** Marie Daclin, Denis-Jean David, Cédric Carbonneil

## Abstract

**Introduction:**

The objective of this assessment was to determine the benefit of using a next-generation sequencing (NGS) gene panel for the clinical management of gastrointestinal stromal tumor (GIST) among patients in routine clinical practice. The aim was to assess the clinical utility of this procedure, the somatic molecular alterations of specific interest, and to define its role in the therapeutic care of patients with GIST.

**Methods:**

The method used for this fast-track assessment were based on: (i) a critical analysis of systematic reviews, meta-analyses, and clinical practice guidelines identified by a systematic literature search; (ii) identification of the level of evidence of molecular alteration clinical actionability as set out by the European Society for Medical Oncology Scale, of targeted therapies included on the list of reimbursable drugs assessed by the French National Authority for Health, or drugs that have compassionate use authorizations issued by the French National Agency for Medicines and Health Products Safety; and (iii) stakeholder consultations and observations by public health institutions.

**Results:**

Assessment of the evidence and data demonstrated that NGS gene panel testing has: (i) superior diagnostic performance for detecting KIT and PDGFRA molecular alterations, compared with Sanger sequencing; (ii) superior diagnostic performance for detecting NTRK1/2/3 fusion, compared with immunohistochemistry; and (iii) evidence of clinical utility of the targeted gene panel considering the benefits provided by targeted therapies.

The composition of the NGS gene panel may be subject to change, in accordance with favorable assessments of new gene alterations. New assessments will be conducted in a dynamic manner in response to developments in scientific knowledge.

**Conclusions:**

The French National Authority for Health deemed that funding the NGS gene panel for GIST was justified for: (i) KIT and PDGFRA genes in cases of locally advanced or metastatic GIST at an intermediate or high risk of recurrence, and in cases of suspected GIST when histology is inconclusive for diagnosis; and (ii) the NTRK1/2/3 gene in cases of refractory or relapsed locally advanced or metastatic pediatric wild-type GIST.

